# Evaluation of the Oxiris Membrane in Cardiogenic Shock Requiring Extracorporeal Membrane Oxygenation Support: Study Protocol for a Single Center, Single-Blind, Randomized Controlled Trial

**DOI:** 10.3389/fcvm.2021.738496

**Published:** 2021-10-11

**Authors:** Stefan Andrei, Maxime Nguyen, Vivien Berthoud, Marie-Catherine Morgant, Belaid Bouhemad, Pierre-Grégoire Guinot, The ECMORIX Study Group, Audrey Martin

**Affiliations:** ^1^Anaesthesiology and Critical Care Department, Dijon Bourgogne University Hospital, Dijon, France; ^2^Anaesthesiology and Critical Care Department, Carol Davila University of Medicine, Bucharest, Romania; ^3^University of Burgundy Franche Comté, Dijon, France; ^4^Cardiac Surgical Department, Dijon Bourgogne University Hospital, Dijon, France

**Keywords:** cardiogenic shock, heart failure, extracorporeal membrane oxygenation, artificial membrane, oxiris, endotoxin/blood, cytokines/blood, continuous renal replacement therapy

## Abstract

**Background:** Veno-arterial extracorporeal membrane oxygenation (VA-ECMO) is the rescue treatment proposed to patients with refractory cardiogenic shock. The VA-ECMO implantation promotes inflammation and ischemia-reperfusion injuries through the VA-ECMO flow, causing digestive mucosa barrier disrupture and inducing translocation of bacterial wall components—Lipopolysaccharides (LPS) with further inflammation and circulatory impairment. LPS is a well-studied surrogate indicator of bacterial translocation. Oxiris membrane is a promising and well-tolerated device that can specifically remove LPS. The main study aim is to compare the LPS elimination capacity of Oxiris membrane vs. a non-absorbant classical renal replacement (RRT) membrane in patients with cardiogenic shock requiring VA-ECMO.

**Methods:** ECMORIX is a randomized, prospective, single-center, single-blind, parallel-group, controlled study. It compares the treatment with Oxiris membrane vs. the standard continuous renal replacement therapy care in patients with cardiogenic shock support by peripheral VA-ECMO. Forty patients will be enrolled in both treatment groups. The primary endpoint is the value of LPS serum levels after 24 h of treatment. LPS serum levels will be monitored during the first 72 h of treatment, as clinical and cardiac ultrasound parameters, biological markers of inflammation and 30-day mortality.

**Discussion:** Oxiris membrane appears to be beneficial in controlling the VA-ECMO-induced ischemia-reperfusion inflammation by LPS removal. ECMORIX results will be of major importance in the management of severe cases requiring VA-ECMO and will bring pathophysiological insights about the LPS role in this context.

**Clinical Trial Registration:** www.ClinicalTrials.gov, identifier: NCT04886180.

## Introduction

Cardiogenic shock is associated with high morbidity and mortality due to impaired tissue perfusion (1). Approximately, 10% of the patients with refractory shock to medical treatment require circulatory support (2). In this case, veno-arterial extracorporeal membrane oxygenation (VA-ECMO) is the bridge-to-recovery treatment of choice. Despite VA-ECMO support, patients with cardiogenic shock have high morbidity and mortality (≥50%) (3). Morbidity and mortality are due to several interconnected mechanisms: tissue hypoperfusion, ischemia-reperfusion phenomenon, and systemic inflammatory response following the cardiogenic shock and VA-ECMO implantation (4–7). These conditions are responsible for multiple organ failure (MOF) and death (1, 8).

The digestive tract mucosa barrier function is affected during the ischemia-reperfusion phase, being a catalyst for systemic inflammation and MOF progression (9, 10). Lipopolysaccharides (LPS) are bacterial wall components that can be found in the intestinal lumen. From animal model and human neonates' studies, it was demonstrated that ECMO-related SIRS may disrupt the intestinal mucosa (11–13), favoring LPS translocation into the plasmatic sector (8, 10, 14). Acute ischemic heart failure also induces intestinal hypoperfusion and intestinal mucosal injury thus LPS translocation (14). LPS translocation promotes systemic inflammation through the activation of toll-like receptors, then it worsens vasoplegia and MOF with further mucosal barrier disruptions entering a positive feedback loop (15). Systemic inflammation with LPS translocation and digestive barrier disruption plays a pivotal role in patient's evolution to MOF and death (12).

The Oxiris filter set has a 3-layer structure with a polyethyleneimine surface-treated and heparin-coated enhanced AN-69 membrane. This particular structure allows for several cytokines and LPS elimination (16). *In vitro* and human studies demonstrated that LPS adsorption decrease the cytokine, systemic inflammation, vasoplegia, with less harmful adverse effects on splanchnic perfusion (16, 17). Furthermore, Oxiris membrane may not be associated with more complications than the usual continuous venovenous hemofiltration membrane (17). The ability of the Oxiris membrane to eliminate cytokine and bind LPS suggests that it might be a potential candidate to control the magnitude of the ischemia-reperfusion-induced inflammation, in order to impair the bacterial translocation and progression to MOF. To our knowledge, there is no published or ongoing clinical trial evaluating the Oxiris membrane in patients with cardiogenic shock support by VA-ECMO.

Our hypothesis is that the early treatment by the Oxiris membrane in patients with cardiogenic shock support by VA-ECMO would allow the removal of LPS and pro-inflammatory cytokines, thus controlling systemic inflammation, vasoplegia, MOF, and death.

## Materials and Methods

### Study Design

ECMORIX is a randomized, prospective, single-center, single-blind, parallel-group, controlled study. It compares the Oxiris membrane vs. the standard RRT care in patients with cardiogenic shock support by VA-ECMO. The study was approved by an independent ethics committee (Ethics Committee No. 2-21-005 id11120, French CPP Sud-Ouest et Outre-Mer 2) and was registered on Clinicaltrials.gov (NCT04886180). The study is conducted at the cardio-vascular ICU of the university-affiliated tertiary hospital of Dijon, France. All patients or their next of kin received written information about the study and gave their written consent to participate. The study design adheres to the requirements of the Medical Research Involving Human Subjects Act and of the Declaration of Helsinki.

The Standard Protocol Items: the study follows the SPIRIT Recommendations for Interventional Trials, and the SPIRIT protocol chronology is presented in Table 1. The first patient was included in May 2021, and the last patient follow-up is planned for June 2024.

**Table 1:** Protocol chronology of the study.

	**ICU admission/ECMO implantation**	**Enrollment**	**Follow-up**					**End of study**
	**H-12 (Day 0)**	**H0 (Day 0)**	**H6**	**24H (Day 1)**	**48H (Day 2)**	**72H (Day 3)**	**Day 7**	**Day 30**
Enrollment validation	X							
Randomization and membrane placement		X						
Clinical examination	X							
Standard analysis set	X	X	X	X	X	X		
Study analysis set	X	X	X	X	X	X		
Clinical and biological data collection		X	X	X	X	X	X	X
Echocardiography		X	X	X	X	X	X	X

*ICU, intensive care unit; ECMO, extracorporeal membrane oxygenation; H, hours before or after enrollment*.

### Study Population

Adult patients are considered for enrolment if they have cardiogenic shock support by VA-ECMO, with an indication for renal replacement therapy (RRT). The inclusion, non-inclusion and exclusion criteria are provided in Table 2.

**Table 2:** Inclusion, non-inclusion and exclusion criteria of the study.

**Inclusion criteria**	**Non-inclusion criteria**
• Age ≥18 years	• Age <18 years
• Family or legal responsible agreement	• Pregnant or breastfeeding patient
• Refractory cardiogenic shock treated with ECMO and requiring RRT	• Severe bleeding under ECMO • No medical insurance
• Inclusion in ≤ 12 h since ECMO implantation	• Patient under court order
	• Patient under legal protection
	**Exclusion criteria**
	• ≤ 24 h of duration of Oxiris or RRT treatment

*ECMO, Extracorporeal membrane oxygenation; RRT, renal replacement therapy; h, hours*.

### Study Intervention

All patients are treated with peripheral venous-arterial femoro-femoral ECMO. Care for patients supported by VA-ECMO is standardized, as previously described (18, 19), and follow patients care practice ESLO guidelines^1^.

Enteral nutrition is started within 24 h of ECMO starting after obtaining hemodynamic stabilization. The enteral nutrition rate follows international guidelines (20). The feeding dose and flow rate is progressively increase on several days (20).

The study treatment consists of either continuous RRT using the Oxiris membrane or an ST150 PrismaFlex membrane for 72 h, respecting the randomization. The continuous RRT is initiated and managed according to the usual practice, only the type of RRT filter membrane is different. The RRT is standardized to continuous veno-venous hemofiltration (CVVH), with a patient dose of 30 ml/kg/h, a predilution of 1/3, and a postdilution of 2/3. The blood flow rate is set between 150 and 350 ml/min to obtain a filtration fraction ratio of 20%. Ultrafiltration rate is set according to patient volemia status. For both groups, the RRT system is incorporated to the VA-ECMO system, as part of routinely process in our ICU.

### Randomization

The 1:1 ratio randomization is performed by the investigator after the patient's enrollment, using a dedicated e-platform made with CleanWeb TM. This process is controlled by individual usernames and newly created passwords for each enrollment. A second checkup for the inclusion, non-inclusion and exclusion criteria is done at this point. Technical support is provided by the Research Methodology Support Unit from our university-affiliated hospital, which also keeps a record of the full randomization process.

### Objectives

The primary objective is to compare the LPS concentration at 24 h between Oxiris membrane vs. a non-absorbent classical membrane (ST150) in patients with cardiogenic shock support by VA-ECMO.

The secondary objectives are (1) to evaluate the effect of Oxiris membrane treatment on the severity of vasoplegic circulatory failure assessed by the vasoactive-inotropic score (VIS); (2) to evaluate the effect of Oxiris membrane treatment on organ failure using the Sequential organ failure assessment (SOFA) score; (3) to evaluate the effect of Oxiris membrane treatment on inflammation and digestive tract barrier disruptor; (4) to describe the Oxiris membrane LPS elimination kinetic profile; (5) to evaluate the Oxiris membrane treatment on the intensive care unit (ICU) length of stay and 30-day mortality.

### Endpoints or Outcome Measures

The primary endpoint is the LPS serum level after 24 h of continuous RRT with Oxiris membrane.

The secondary endpoints are: (1) the VIS score [defined using the formula: Dopamine infusion rate (mcg/kg/min) + Dobutamine infusion rate (mcg/kg/min) + 100 x Epinephrine infusion rate (mcg/kg/min) + 10 x Milrinone infusion rate (mcg/kg/min) + 10,000 x Vasopressin infusion rate (units/kg/min) + 100 x Norepinephrine infusion rate (mcg/kg/min)]; (2) the SOFA score at day 1, 2, 3 and 7; (3) Serum levels kinetic of cytokines (IL1, IL6, IL 4, IL10, TNF-alpha, interferon gamma, MCP1), CRP, procalcitonin, I-FABP, GLP-1, zonuline, citrulline from RRT initiation, to 6 h after initiation, day 1, day 2, and day 3 of treatment; (4) LPS plasma concentration, (5), ICU length of stay and 30-day mortality. Patients' follow-up is 30 days after initiation of VA-ECMO.

Serial standard usual blood analysis set is drawn at several time points: at the enrollment moment (before RRT membrane placement), after 6, 24, 48, and 72 h of continuous RRT. The study-specific blood analyses are performed at the same time moments. The reference time (T0) is the start of RRT for all group (control and intervention).

At each time point, one EDTA tube of 5 ml is taken via the arterial catheter, and 2 sets of EDTA tubes of 5 ml and are taken on the RRT circuit (one before and one after the filter membrane).

### Blinding

All data will be collected by a non-managing investigator blind to treatment therapy. All analyzes will be performed blind to treatment therapy.

### Data Collection

Data will be collected and recorded on eCRF. All patient-related data are anonymously and numerically coded.

Data are as follows: age, sex, weight, SOFA score, SAPS II score, hospital and ICU admission reason, past medical history, comorbidities, VIS score, sedation drugs (type and infusion rate), hourly urinary output, amount of fluid, fluid balance, respiratory parameters (tidal volume, respiratory rate, positive end-expiratory pressure, inspiratory O_2_ fraction, plateau pressure), enteral nutrition (flow rate and kcal/kg/d), hemodynamic (heart rate, arterial blood pressure, pulse pressure), transthoracic echocardiographic evaluation, VA-ECMO parameters [flow rate, FmO_2_, O_2_ flow rate (l /min)].

All patients treated with VA-ECMO have daily transthoracic echocardiography until ECMO withdraw. Cardiac echocardiographic parameters are anonymously recorded at each time points and analyzed in a second time by an investigator non-involved in the patient's clinical management and blind to allocation treatment. Echocardiography is performed by an investigator with cardiac ultrasound certificate. Echocardiography parameters are: right and left cardiac chamber size, right and left systolic cardiac function, left and right diastolic parameters (mitral inflow, mitral annular velocities, tricuspid inflow, tricuspid annular velocities), cardiac output, inferior vena cava diameter, portal flow. The analysis will be performed offline in a simple blind manner.

### Assessment and Reporting of Adverse Events

Adverse events will be recorded in detail during both the follow-up periods. The reporting and documentation of adverse events will be classified as IMT-related or non-IMT-related. This safety analysis will be conducted at the end of our research.

### Statistics

#### Sample Size Calculation

Based on a LPS mean value of 110 ± 40 pmol/l (21), 34 patients should be randomized, 17 in each treatment arm will be necessary to demonstrate a decrease over 35% (16), with a bilateral alpha risk of 5% and a power of 80%. Assuming that 15% of patients would not be evaluable due to early death, we fixed the sample size to 40 patients, 20 patients in each treatment arm.

#### Data-Analysis Plan

The data will be analyzed in intention-to-treat and per-protocol. The variables will be presented as mean ± SD, or as median [25–75% IQR], as appropriate. The treatment groups will be presented comparatively after the randomization. The qualitative variables will be compared using the Chi-square test or Fisher's exact test. The quantitative variables will be compared using the Student's test or a non-parametric test, as appropriate. Longitudinal data will be processed using mixed-models. The normality will be checked graphically, with histograms and QQ plots and using the Shapiro-Wilk normality test.

The evaluation of the primary endpoint will be considered as a comparison of means. The statistical analysis will be performed using R Studio software. A *p* < 0.05 will be considered statistically significant.

## Discussion

The results of this study will bring insights into the potential use of the Oxiris membrane in managing cardiogenic shock supported by VA-ECMO. Furthermore, the particular focus on the LPS serum levels profile will provide a better understanding of the mechanisms of therapeutical benefit of Oxiris membrane.

Blood purifications is a subject of rising interest, and some authors proposed a rationale for blood purifiers use to control VA-ECMO-induced systemic inflammation (22). However, the use of cytokine absorbent filters is still controversial and lacking scientific evidence. The Cytosorb membrane (Cytosorbents, NJ, USA) has been studied in various settings (23–26). The Cytosorb membrane is able to eliminate a large diversity of cytokines, but also other plasmatic molecules like DAMP, PAMP, complement factors (27–29). However, Cytosorb did not improve clinical outcomes when used during the cardio-pulmonary bypass (30). Moreover, in patients suffering of SARS-Cov2 supported by ECMO, Cytosorb was unable to decrease interleukin 6 concentrations, and was associated with higher mortality (31). These findings suggest that inflammation is insufficiently controlled by the Cystosorb membrane and one hypothesis is that an alternative pathway might be involved.

The Oxiris membrane (Baxter Investment Co., Ltd., Shanghai, China) is another adsorbing membrane that has been less studied. One important difference between those 2 membranes is that the Oxiris membrane is able to remove both interleukins and LPS (16, 28). Those properties seem to promote hemodynamic stabilization and even organs failure improvement during states of sepsis-related circulatory failure (17, 32–35). Because LPS might play a pivotal role in enhancing inflammation during the ischemia-reperfusion phase, the additional LPS removal properties of the Oxiris membrane by reducing the triggering of the TLR-4 pathway could provide a clinical benefit. As this clinical benefit has not yet been demonstrated in our population those hypotheses remain speculative and randomized trials are needed to provide robust answers.

Indeed, the literature on the subject is parse and mainly performed in human neonates or animals with immature gastrointestinal tract and/or different immune system (7, 11). No study has evaluated the effect of retrograde VA-ECMO flow on intestinal perfusion and intestinal barrier. Despite cardiogenic shock is associated with intestinal translocation and high level of LPS, few studies have demonstrated this point in cardiogenic shock supported with VA-ECMO. In addition, our study doesn't include a control group of cardiogenic shock patients without VA-ECMO support. All these points could be a limit of the study. Nevertheless, by focusing on LPS removal kinetic our study will allow clarifying for the first time whether the Oxiris device lowers the LPS burden in refractory cardiogenic shock supported by ECMO (10–12).

One aspect of particular interest in our population is the predictability of the timing of the injury that allows early treatment before the irreversible consequences of ischemia-reperfusion. This fact represents an advantage comparing to medical conditions-induced cytokine storm. Nevertheless, the right initiation timing during VA-ECMO is debatable and to be determined (22). One should also consider the more extensive ICU discussion about the adequate RRT initiation moment (29). We chose to enroll the patients no later than 12 h after VA-ECMO implantation in order to anticipate the irreversibility of ischemia-reperfusion injury.

The adding of an extracorporeal circuit seems to be clinically tolerated, but it is difficult to propose it for patients who do not need RRT. Because Oxiris membrane is used on VA-ECMO, RRT blood flow rate may be low and the LPS clearance rate may be insufficient. Broman et al. demonstrated that RRT with a mean blood flow rate of 132 ± 69 ml min^−1^ is able to significantly decrease endotoxin level (17). This blood flow rate is below the blood flow rate in our study. The main concern is the risk of hemorrhage due to vascular access, extracorporeal-induced coagulopathy, and the immune reactions to the heparin-coated circuit. Regarding this concern, the patients on VA-ECMO are already at risk with a larger extracorporeal circuit, large cannulae for vascular access and already on heparin anticoagulation. The addition of the RRT circuit can be performed on the VA-ECMO circuit, as already shown (36).

In conclusion, the study results will be of significant importance in the management of refractory cardiogenic shock support by VA-ECMO, and will bring pathophysiological insights about the intestinal barrier and LPS role in this context.

## Ethics Statement

The studies involving human participants were reviewed and approved by Ethics Committee French CPP Sud-Ouest et Outre-Mer 2, No. 2-21-005 id11120. The patients/participants provided their written informed consent to participate in this study.

## Author Contributions

P-GG, MN, and VB contributed to the conception and design. P-GG, MN, SA, and M-CM searched the associated data. SA, MN, and P-GG drafted the manuscript. BB provided the supervision support. MN performed data analysis. All authors contributed to the critical revisions and final approval of the manuscript.

## Funding

The authors performed this study in the course of their normal duties as full-time employees of public healthcare institutions.

## ECMORIX Study Group

Audrey Martin, Mohamed Radhouani, Tiberiu Constandache, Sandrine Grosjean, Pierre Voizeux, Emel Rafrafi, Chloe Bernard, Saed Jazayeri, Ghislain Malapert, Olivier Bouchot.

## Conflict of Interest

The authors declare that the research was conducted in the absence of any commercial or financial relationships that could be construed as a potential conflict of interest.

## Publisher's Note

All claims expressed in this article are solely those of the authors and do not necessarily represent those of their affiliated organizations, or those of the publisher, the editors and the reviewers. Any product that may be evaluated in this article, or claim that may be made by its manufacturer, is not guaranteed or endorsed by the publisher.

## Footnotes

^1^ https://www.elso.org/Resources/Guidelines.aspx
